# Purchasing and Using Personal Emergency Response Systems (PERS): how decisions are made by community-dwelling seniors in Canada

**DOI:** 10.1186/s12877-015-0079-z

**Published:** 2015-07-11

**Authors:** Alexandra C. McKenna, Marita Kloseck, Richard Crilly, Jan Polgar

**Affiliations:** Graduate Program in Health and Rehabilitation, Faculty of Health Sciences, The University of Western Ontario, 1151 Richmond Street North, London, ON N6A 5B9 Canada; School of Health Studies, Faculty of Health Sciences, The University of Western Ontario, HSB 222, Arthur and Sonia Labatt Health Sciences Building, 1151 Richmond Street North, London, ON N6A 5B9 Canada; Department of Medicine, Division of Geriatric Medicine, The University of Western Ontario, Parkwood Hospital, 801 Commissioners Road East, London, ON N6C 5 J1 Canada; School of Occupational Therapy, Faculty of Health Sciences, Western University, Room 2549, Elborn College, 1201 Western Road, London, ON N6G 1H1 Canada

**Keywords:** Personal emergency response systems, Grounded theory, Decision-making process, Oldest-old

## Abstract

**Background:**

As the demographic of older people continues to grow, health services that support independence among community-dwelling seniors have become increasingly important. Personal Emergency Response Systems (PERS) are medical alert systems, designed to serve as a safety net for seniors living alone. Health care professionals often recommend that seniors in danger of falls or other medical emergencies obtain a PERS. The purpose of the study was to investigate the experience of seniors living with and using a PERS in their daily lives, using a qualitative grounded theory approach.

**Methods:**

Five focus groups and 10 semi-structured interviews, with a total of 30 participants, were completed using a grounded theory approach. All participants were PERS subscribers over the age of 80, living alone in a naturally occurring retirement community (NORC) with high health service utilization in a major urban centre in Ontario. Constant comparative analysis was used to develop themes and ultimately a model of why and how seniors obtain and use the PERS.

**Results:**

Two core themes, unpredictability and decision-making around PERS activation, emerged as major features of the theoretical model. Being able to get help and the psychological value of PERS informed the context of living with a PERS.

**Conclusions:**

A number of theoretical conclusions related to unpredictability and the decision-making process around activating PERS were generated.

## Background

As seniors age, they may face more obstacles to maintaining their independence. Particularly with those 80 years of age or older there are many challenges to staying in their own home. With advancing age, environmental, mobility and other supports that make activities of daily living easier and safer become increasingly important and the dangers of not being able to get help in a medical emergency become more serious. Personal Emergency Response Systems (PERS) are medical alert systems designed to aid individuals living alone in the community to summon help when emergency situations occur. PERS are designed to be worn 24 hours a day, seven days a week so seniors can get help if needed [1].

PERS are designed in the form of a necklace or bracelet. A typical PERS has three main components [1, 2]. A radio transmitter in the form of a button on the necklace or bracelet is pressed by the subscriber when in distress. This activates a communicator attached to the user’s phone line which acts as a speakerphone between the senior and the emergency response centre. The emergency response centre responds by dispatching an ambulance or contacting the responder identified by the senior. There is an initiation fee and monthly fee which are paid by the individual subscribers but there is no extra cost for activating the system. The system can be ordered by individuals for themselves, by family members, or by professionals for their clients/patients but the PERS is not considered a medical devise and is paid for by the subscriber.

Since their invention, PERS have been marketed as a way for seniors to maintain their independent lifestyle. High rates of satisfaction have been found with PERS users and many families of subscribers reported having peace of mind [3–6]. Subscribers to PERS have also been found to have a decrease in hospital length of stay [7, 8] which has implications for health care cost savings, although no explanation for this was provided by the authors. The fear of falls is cited as a major reason for seniors getting a PERS, and early research reported subscribers of PERS had a reduced fear of falling [3, 4].

There have, however, been inconsistencies in the literature regarding the effectiveness of PERS. In a randomized control trial in Alberta, Canada, the impact of PERS on anxiety and fear of falling was investigated among older adults discharged from hospital [9]. There was no statistically significant change in anxiety or fear of falling in PERS users compared to non-users [9]. Additionally, while Porter’s qualitative studies [10–12] emphasized positive aspects of PERS, such as certainty of getting help, her studies also highlighted negative aspects, such as unexpected responder visits or uncertainty over whether to push the button. Whatever the overall value of PERS, there remain many questions regarding why people do or do not subscribe, and having obtained it, why they might or might not use it [6].

The purpose of this study was to investigate the experience of seniors living with and using a PERS in their daily lives, using a qualitative, grounded theory approach [13]. The following research questions were addressed: How do individuals become PERS users? Does the PERS operate the way seniors want or expect it to? Under what circumstances do seniors view it acceptable to push the PERS button?

## Methods

To explore the process of an older adult living with and using a PERS, a grounded theory approach was used. Grounded theory recognizes that events and actions happen within a social context, which is complex and affected by many different forces such as political, gender, racial and cultural issues [13]. This research project sought the reality of using and living with a PERS for older individuals living alone, thus Corbin and Straus’ [13] epistemological framework, which incorporates pragmatism and symbolic interactionism, was used. The pragmatic perspective places value on knowledge created through actions and interactions, grounded in a social environment, while symbolic interactionism advocates for human beings as dynamic objects, acting and interacting and creating the knowledge which pragmatists value.

### Participants

Purposive sampling [14] was used to recruit participants from a naturally occurring retirement community (NORC) with high health service utilization in a major urban centre in Ontario. This community consists of 12 privately owned apartment buildings housing 2730 seniors (mean age = 79, *SD* = 9.53) and 64 local businesses housed in a public mall adjacent to the apartment buildings. A previous survey showed that 54 % of the population in this community were over 80 years of age. Inclusion criteria included: (i) being 80 years of age or older, (ii) living alone, (iii) being a current PERS subscriber, (iv) fluency in English, and (v) being able to carry on a conversation with, and answer questions posed by, the primary researcher. To recruit participants, notices were posted on public boards in the mall which serves this community, as well as on notice boards in common areas in the 12 apartment buildings. The same advertisement was also placed in the monthly community newsletter provided to residents of the NORC. Several gatekeepers in the NORC were also contacted. These included volunteer community leaders who worked with the primary researcher to promote the study at a townhall meeting. In addition, the coordinator of the main PERS provider within the NORC agreed to contact subscribers to promote the study.

All potential participants were given a letter of introduction and provided written informed consent to participate in the study. This study was approved by the Research Ethics Board at the University of Western Ontario.

### Data collection

This study used five focus groups and ten semi-structured individual interviews to explore the use of PERS by community-dwelling older individuals. Traditionally focus groups take place with 6-12 participants to gather a wide breadth of information. It has been found that with older adults, smaller focus groups of 6-8 participants are better geared to encourage discussion while still gathering rich data. Saturation is reached when no new information emerges for a particular concept [13]. Previous experience with this study population demonstrated that saturation was reached by the end of two focus groups. To ensure saturation, three additional rounds of focus groups were conducted. Semi-structured interviews, with 6-10 participants, are routinely used to probe more deeply into emerging themes identified in group discussions and to ensure enough in-depth data is collected. In this study focus groups were completed first and used to generate ideas for the interview and to fine-tune interview questions. By performing focus groups prior to interviews, ideas and topics of interest that emerged from the focus groups were flagged and discussed in-depth during the semi-structured interviews. Participants in the interviews were different from those participating in the focus groups. 

A total of five focus groups were conducted with 3-6 participants per focus group. Focus groups ranged in length from 60-90 minutes. A semi-structured question guide was used to focus group discussion. Participants were asked how they decided to subscribe to a PERS, how and when they wear a PERS, how they decide to push the button, how the PERS influences their independence, and how well their PERS works for them. Based on the emerging themes and categories from the focus groups, two questions and three probes were added to the individual interviews to explore the issues more deeply. The questions focused on decision-making and PERS education and instruction, while the probes were added to gather more detailed information on daily routine with PERS, experiences with PERS, and the relationship between PERS and independence. Individual interviews lasted 30-75 minutes. The primary researcher conducted the focus groups with a member of the research team taking notes regarding such things as tone and body language. All focus groups and interviews were recorded using a digital voice recorder. A short socio-demographic survey was distributed to participants upon completion of the focus groups and interviews to capture participant characteristics.

Focus group and interview recordings were transcribed by the primary researcher. All sessions were recorded using a digital recorder, then coded using NVivo software.

### Data analysis

Following transcription, data were analyzed using a constant comparative method [13]. Constant comparison is a grounded theory data analysis technique whereby data analysis takes place concurrently with data collection after every focus group and interview. As themes emerged, they were compared to those from previous focus groups and interviews. The data analysis procedure included open, axial and selective coding, using the qualitative coding program NVivo 7. Open and axial coding enabled comparison, the creation of categories and themes and identified relationships. For example, open codes such as *freedom to make own decisions*, *control over matters*, *methods of transportation* and *keeping busy*, were all grouped under the contextual category of *do what I want, when I want*, a major contextual component of independent living among the participants. Open coding began with line-by-line coding of each transcript. More than 100 codes were created, which were eventually grouped into less than 20 nodes or categories. Corbin and Strauss’s [13] paradigm was used to organize categories within the framework of: causal conditions, phenomena, context, intervening conditions or consequences, and actions/interactions/strategies. Once the researcher had considered where the categories best fit, selective coding began and the different categories were looked at together as part of the bigger picture. Emerging themes were used to create a logical story that reflected participants' experiences and perceptions. A rich contextual picture emerged of living independently as a senior, as well as a rich context of living with a PERS.

To ensure quality and rigour, Lincoln and Guba’s [15] trustworthiness criteria were used. The trustworthiness criteria were created to parallel the traditional scientific values of validity and reliability. Credibility was ensured by triangulation, member checking and peer debriefing. Triangulation took place by using two data collection methods: focus groups and interviews. In addition coding conducted by the primary researcher was compared with independent coding conducted by research team members. Through member checking with focus group participants, comments and experiences were confirmed. Debriefing with other members of the research team also occurred as part of thematic development to ensure sound analysis. To ensure dependability and confirmability, memoing was undertaken in accordance with grounded theory method and a reflexive journal was maintained throughout the study to record the thoughts and feelings of the primary researcher. The reflexive journal also provided transparency regarding the beliefs and feelings of the primary researcher so as not to bias the study results. To ensure transferability, thick descriptions were used, including quotes from interview and focus groups [16] to fully support the emergent model and to deliver a rich account of the participant’s context and experiences. Socio-demographic data were analyzed using descriptive statistics.

## Results

A total of 30 participants took part in this study. Five focus groups were held with 3-6 individuals per focus group, for a total of 20 focus group participants, with an average age of 88.4 (±5.20 SD). Focus groups were followed by 10 individual semi-structured interviews. The average age of interview participants was 88.9 (±3.60 SD). A complete description of the socio-demographic characteristics of focus group and interview participants is provided in Table 1. In general, study participants were quite active and independent, with 56.7 % leaving their home nearly every day. Self-rated independence was high, as more than half the participants reported being completely or very independent. Self-perceived health varied, with 76.7 % of all participants reporting good, very good or excellent health.

**Table 1:** Socio-demographic characteristics of PERS subscribers

Characteristic	Focus groups (*N* = 5)	Interviews (*N* = 10)
No. participants	*n* = 20	*n* = 10
Mean age (years)	88.4 (SD ± 5.20)	88.9 (SD ± 3.60)
Gender		
Male	20 % (*n* = 4)	-
Female	80 % (*n* = 16)	100 % (*n* = 10)
Marital status		
Single	5 % (*n* = 1)	10 % (*n* = 1)
Widowed	85 % (*n*=17)	80 % (*n* = 8)
Married	5 % (*n* = 1)	-
Separated	-	-
Divorced	5 % (*n* = 1)	-
Common-law	-	-
Education		
Public school	30 % (*n* = 6)	10 % (*n* = 1)
High school	45 % (*n* = 9)	30 % (*n* = 3)
College (diploma)	10 % (*n* = 2)	30 % (*n* = 3)
University (Bachelors)	5 % (*n* = 1)	10 % (*n* = 1)
University (Masters)	5 % (*n* = 1)	-
University (PhD/MD)	-	-
Other education	5 % (*n* = 1)	20 % (*n* = 2)
Perceived Health^a^		
Excellent	5 % (*n* = 1)	10 % (*n* = 1)
Very good	20 % (*n* = 4)	30 % (*n* = 3)
Good	50 % (*n* = 10)	40 % (*n* = 4)
Fair	25 % (*n* = 5)	20 % (*n* = 2)
Poor	-	-
Mean Self-Rated Independence^b^		
Completely independent	10 % (*n* = 2)	20 % (*n* = 2)
Very independent	25 % (*n* = 5)	70 % (*n* = 7)
Somewhat independent	25 % (*n* = 5)	10 % (*n* = 1)
Very little independence	5 % (*n* = 1)	-
Not at all independent	-	-

^a^Self-rated health ranged from 1(excellent) to 5 (poor)

^b^Self-rated independence ranged from 1 (completely independent) to 5 (not at all independent)

Not all numbers add up to 100 % because of non-responders in some categories

 PERS details regarding subscriber use of and satisfaction with the device are outlined in Table 2. Subscription time ranged from less than one year to 10 years, with an average subscription of 3.3 years. Eight of the participants had pressed the button in 25 different situations. Of the 25 instances in which the button was pressed, only nine of those situations resulted in a trip to the hospital. Of the 30 participants, all subscribed to a PERS, but only eight had actually pressed the button in emergency situations. Of those, reasons for pushing included heart attacks, falls, diabetic episodes, and inability to get out of their tub. There were also situations where a PERS could have been activated but participants simply forgot about the device they were wearing or forgot to push the button when an emergency situation arose. Other situations where a PERS could have been activated included one participant who decided to “manage” the situation on her own rather than disturb her responders. In addition to medical emergencies, break-ins or fires were also situations when the button could be pressed but had not occurred with these participants. One-fifth (20 %) of the participants reported their machine having gone off by accident. Overall, 76.7 % of participants reported being usually satisfied or extremely satisfied with their PERS subscription. In addition, 90 % of the participants strongly or somewhat agreed to the statement, “Connect Care (a PERS provider) helps me maintain my independence”.

**Table 2:** PERS Subscribers’ use of and satisfaction with device

Characteristic	Focus groups (*n* = 20)	Interviews (*n* = 10)
Average length of subscription (years)	m = 3.68	m = 2.58
Who signed senior up for PERS		
I signed up	45 % (*n* = 9)	90 % (*n* = 9)
Family member	25 % (*n* = 5)	10 % (*n* = 1)
Friend or neighbour	15 % (*n* = 3)	-
Doctor/health professional	-	-
Other	10 % (*n* = 2)	-
How often is device worn when home?		
Every day, all the time	90 % (*n* = 18)	80 % (*n* = 8)
Every day, but just for a few hours	-	-
A few hours every week	-	-
Not at all	10 % (*n* = 2)	20 % (*n* = 2)
How often is device kept within arms reach?		
Always	75 % (*n* = 15)	90 % (*n* = 9)
Usually, but not always	15 % (*n* = 3)	-
Sometimes	10 % (*n* = 1)	10 % (*n* = 1)
Almost never	-	-
Never	-	-
During day but not at night	-	-
How many times have you pushed (in a non-test situation)?	23 (*n* = 6)	2 (*n* = 2)
How many of those (total) instances resulted in trips to hospital?	8 (*n* = 5)	1 (*n* = 1)
Has the PERS ever activated by accident?		
Yes	25 % (*n* = 5)	10 % (*n* = 1)
No	50 % (*n* = 10)	90 % (*n* = 1)
No response	25 % (*n* = 5)	-
Satisfaction with your PERS^a^	m = 5.26 SD ± 1.05	m = 5.0 SD ± 1.00
To what extent do you agree or disagree with the statement: “ConnectCare helps me maintain my independence”^b^	m = 1.15 SD ± 0.37	m = 2.5 SD ± 0.97

^a^Satisfaction with PERS was rated on a Likert-type scale of 1 (extremely dissatisfied) to 6 (extremely satisfied)

^b^Rated on a Likert-type scale of 1(strongly agree) to 5 (strongly disagree)

Not all numbers add up to 100 % because of non-responders in some categories

Thematic saturation was reached by the end of the five focus groups with no new topics emerging. No new topics emerged from the 10 individual interviews, but interviews did provide greater depth into the themes. Focus group and interview data were initially analyzed individually, but as no major thematic differences occurred between the two methods, the data were collapsed into one data set for analyses.

Two core themes emerged: unpredictability and the decision-making process of when to push the button. Underlying the causal and contextual conditions is the core theme of *unpredictability* – the day-to-day instability in health and function characteristic of advanced old age, perceived risk of a medical emergency and the unpredictability and necessity of being able to get help while living alone. “I thought it was a good idea to have it (PERS) around ‘cause you never know, I have a bad heart, it can go anytime” (Participant M(male)21). The decision-making process (second core theme) emerged when unexpected stressful or emergency situations actually occurred.

### Theme 1: Unpredictability within the context of isolation

Causal conditions that influenced participants to subscribe to a PERS had social, psychological and physical components. Most participants reported they had decided independently to subscribe to a PERS, but most also reported social influences, such as encouragement by a concerned family member, friend or social representative (Veteran’s Affairs Canada representative). Past physical events such as falls, heart attacks and seizures also influenced participants' decisions to subscribe. “Well I did it myself. But I had been ill. And uh, it really probably at that point wasn’t safe for me not to have some kind of protection” (Participant F(female)22). In addition, some seniors had heard about a friend’s bad experience, where a neighbour was unable to get up for days and was unable to call for help. “You hear about people who fall and then can’t get help and they lay there for sometimes hours, but it just scares you when you think that could happen” (Participant F14). The theme of unpredictability clearly plays a large role in influencing seniors to subscribe to a PERS. The psychological pressure of seeking peace of mind in the face of unpredictability was very important to participants.

They (children) know I’m on my own completely, and I am at the end of the hallway…I’ve just got one neighbour, and I don’t know who that is. I’m a long way, the people next door to me are way at the other end of the floor, so that if I fell, I could be lying there for ages before anybody heard me, without this Connect Care (PERS). (Participant F17)

This theme also incorporated situations where participants heard about a friend’s bad experience, and participants became anxious about getting help for themselves in a similar situation.The underlying concern appears to be fear of the unpredictable event while being isolated from help. PERS was seen as a way to cope with this unpredictability.

Within Theme 1 two main contextual areas became evident during focus groups and interviews: (i) the desire to live independently and retain control over one’s life, and (ii) the necessity of living with a PERS to remain independent by reducing fear of the unpredictable. Under the first contextual area there was a *psychological value* associated with having a PERS. Subscribers reported feeling a sense of security or peace of mind, which played a supportive role in living independently, and was a main part of living with the device. The contextual and causal conditions are all set against a background of *unpredictability,* as participants could not be sure when an emergency or fall might occur. Also within this context were *self-reliance* and being able to *do what I want, when I want*. Self-reliance referred to the seniors’ ability to look after their own needs, “Being able to look after yourself. Taking care of your own apartment. Getting your own meals” (Participant F62). Do what I want, when I want refers to the freedom to do activities as they please. For example, “You also, also now, can do what you want, when you please. It’s your decision” (Participant M21). Seniors appeared to fear the loss of self-reliance and self-sufficiency, and consequently, the loss of their independence and freedom.

I dread just being an ill person who can’t cope with daily looking after yourself, yeah…The last thing I want to do is lose my independence and be an invalid, it’s my biggest fear. I’m speaking very frankly. (Participant F14)

During axial coding, it became clear that many participants' comments and descriptions around independent living were related to self-reliance and ‘doing what I want’. Thus, they were grouped under the over-arching category of control over daily life in independent living.

The second contextual area, the necessity of living with a PERS, incorporated two main responsibilities, *daily routine* and *keeping the PERS working*. In discussions with participants in both focus groups and interviews, it became clear that participants had developed many routines around their PERS. Many participants reported taking their PERS off while bathing or sleeping, but kept it close by on the toilet seat or night table. With regard to leaving their apartments, response was mixed between wearing it outside and leaving it at home.

If I go out for the day I don’t wear it, because it’s only within a certain distance of your box that it works, so uh, no, if I’m going out for the day I don’t wear it. (Participant F14)

As part of their regular routines, PERS subscribers were concerned with keeping their device working and conducted monthly test calls. The PERS companies suggested subscribers make a habit of calling on the same day each month, “So you know its active. You know its working” (Participant M41).

Most participants also knew where the device was when they weren’t wearing it and had places in the home where they would leave the necklace or bracelet when not wearing it, to easily find the device and put it back on.

### Theme 2: The decision-making process – activating PERS

The second core theme, the decision-making process of when to push the button, became evident once an unexpected stressful or emergency situation occurred and other options such as being able to reach the phone were not possible. Almost all participants had been in situations where they considered activating their PERS. This decision-making process was structured by intervening conditions that influenced the activation interaction of PERS, in this case, a need to get help. Intervening conditions influencing the decision-making process around activating the PERS were: awareness and accessibility, acceptability, self-diagnosis and seriousness, and the involvement of others. These conditions are discussed below.

#### Awareness and accessibility

To enter the decision-making process, individuals first had to be aware of their PERS device, and then had to have the ability to press their button if required. Three of the participants had medical emergencies in which they simply forgot about their PERS. Participant F15 had a heart attack, followed by a fall, and then another heart attack. She recounts, “Listen, the button didn’t even concern me, the button didn’t even come into my mind. All I knew I was in trouble. That’s all I knew.” Other participants had concerns that if they were unconscious or the necklace fell behind them and was out of reach, they would be unable to press the button.

#### Acceptability

If individuals are aware of their PERS button and able to push it, then they must decide whether to use it. At this point, there are several things to consider. First, the situation must be acceptable to the individual to push the button – most participants felt they would push the button for any emergency if it was serious enough, but generally it was considered for medical purposes.

Well, I wouldn’t (press the button in non-medical situation) because that’s not what you’re supposed to do. I mean, they’re not involved with my having a fire or any other emergency…well I suppose they would be, but, it would only be if I couldn’t get it (fire) out myself. (Participant F10)

Suppose a caller come to your door, try to sell you something, try to push their way in or something. Well, all you do is press that thing and immediately you get word “house call in progress”, and that would frighten the guy and he takes off. I’ve thought of that many a time. (Participant M1)

#### Self-diagnosis and seriousness

If the situation was medical in nature, many participants talked about performing a self-diagnosis and evaluating the seriousness of the situation. Participant F2 explained how she decided to press her button:

I had chest pains, I thought it was…makes you burp (indigestion) you know, so I waited twenty-four hours, even went to bed. I thought those pains would be gone by morning. They weren’t. So, I pressed the button, and I told them I was having chest pains. I think they must have flew here! I had two heart attacks.

The seriousness of the situation would dictate the amount of internal debate around whether to press. “You think, yes, no, and you’re thinking, well you’ll wait for a little while, see if it gets better” (Participant F41). Participants said they would not hesitate to press the button in a serious situation. However, if the seriousness of the situation was in question, participants reported that they would hesitate. Some participants reported they would feel embarrassed if they pushed the button for a trivial reason.

#### Involvement of others

The issue of involving others was raised by participants over concerns of disturbing or bothering responders:

I didn’t (push the button). Because I thought, I can manage on my own. My responders are …they live about half an hour’s drive from where I am, so I didn’t want to disturb them, lets put it that way, and so things calmed down. (Participant F54)

Others felt it would be unfair not to call the family and let them know about emergency situations. “I think its only fair to let family know…you never know how critical it is” (Participant F15). Involvement of friends or family was also a matter of timing:

It isn’t a question of not wanting them because we’re all family, and if there’s a need to call one of them…But things like emergency, is not always convenient to call them. When having this Connect Care is much faster and more definite by the time they would get here, maybe important time would be lost. (Participant F12)

To enter the decision-making process, the individual must first be conscious and aware that they are wearing their necklace. Once the decision-making process is entered, different considerations of acceptability, self-diagnosis, seriousness, and the involvement of others are reflected upon internally, in no particular order. On the contrary, participants seemed to contemplate many of these considerations simultaneously, for example, by weighing the benefits of getting help with a minor problem compared to the disruption caused to responder’s lives.

The decision-making process is complex and can have consequences for the contexts of day to day living and living with a PERS, as well as for future interactions with the PERS. For example, if an individual activates their PERS during a heart attack and an ambulance comes quickly to their home, they may feel they are indeed protected living independently in their home and wear their PERS at all times when in the home. Alternatively, if someone chooses to endure a stressful situation until it gets better, then they may not perceive their PERS as useful or helpful, and may in the future leave their device on their nightstand.

## Discussion

This study adds richness and depth to the context of situations faced by individuals in advanced old age on a day-to-day basis, the experience of living with a PERS, and the decision-making process around PERS use. This study points towards the concept of older individuals being aware of the unpredictable nature of their health, the threats this brings to their continued independence (living alone), and the use of a PERS to provide a way of controlling or managing the unpredictability. In addition, the study has shed some light on the decision making process, that is, when to activate the device, which is a critical part of the process, and the ambiguity many feel about doing so.

A primary contribution of this study is the development of a theoretical model of living with a PERS, as constructed through qualitative data analysis. This model provides a useful framework for understanding the context and experiences of PERS subscribers. Although PERS literature has, in a limited way, explored the experience of having a PERS [11] and has looked at intentions around using a PERS [12], this study is unique in that the theme of unpredictability emerged as a main contextual condition from the perspective of individuals in advanced old age, in which unexpected health or functional declines can result in serious adverse outcomes. Against this backdrop, PERS plays an important role in being able to get help, and provides subscribers with feelings of security, comfort and/or peace of mind, introducing an element of control into a life characterized by unpredictability.

The present study holds many similarities to prior qualitative research on PERS [10–12, 17, 18]. Porter’s [12] phenomenological study of eight frail women living alone explored the experience of having a PERS and found one of the key components of the context of living with a PERS was “being certain that they can get help quickly” while living alone. The present research demonstrates that being able to get help, against the backdrop of unpredictability, was a main theme in deciding to subscribe to the PERS, as well as a main part of the participant’s experience of living with the device.

### PERS and unpredictability

Unpredictability sets the stage for PERS use. The participants in this study used strategies, such as PERS, to maintain their independence and minimize the risk of the unexpected. Unpredictability is also seen in the PERS literature regarding hospital utilization. Three studies looking at health care utilization rates [7–9] all found that having a PERS did not significantly affect emergency department visits. This theoretical model clearly shows us that having a PERS will not prevent the unexpected stressful events from occurring, but will help get help to the individual quickly, which in turn leads to better health outcomes.

#### Theoretical conclusion 1

With increasing frailty, unpredictable situations commonly occur and risk is always present. Findings from this study highlight the critical role of unpredictability in the day-to-day lives of very old individuals. From these findings, it is proposed that from the perspective of older individuals, frailty is personalized and operationalized as unpredictability and perceived risk. For the subscriber, a PERS is seen as a way of managing unpredictability.

### PERS and the decision-making process

The decision to use the PERS, once obtained, is influenced by an individual’s perception of their risk and is a personal matter. The perception of others of the individual’s risk is of less importance. While PERS does not alter functional ability or alter one’s physical environment, PERS may provide a ‘buffer’ against the stress of unexpected emergency situations. The decision-making process that emerged from these risk situations can be considered a form of a problem-focused coping response [19]. When faced with the stressful situation, the decision-making process and the intervening considerations come into play, a cognitive appraisal of the situation is made, and the coping response (to press or not press) is chosen based on that decision-making process. Results showed that in situations where the level of seriousness was in question, participants were more hesitant to push their button. Participants had concerns about involving others (responders, ambulance staff) or potentially embarrassing themselves for pushing for a non-serious reason. The concerns of the subscriber about inconveniencing their responder led to situations where there was a delay in obtaining help when it was required, which is clearly counter-productive and may increase the likelihood of an adverse outcome.

#### Theoretical conclusion 2

PERS use is influenced by an individual’s level of perceived risk and who initiated the subscription to the device, for example, the users themselves, a daughter or veteran’s affairs representative. The daily routines around wearing PERS varied greatly between individual subscribers, with some choosing to leave it in a specific room while others wore it all the time. Other assistive technology literature reports that devices are more likely to be abandoned if they are purchased by someone besides the user [20–22]. Relevant to this study, if the PERS was purchased for someone who did not perceive risk or unpredictability as impacting their independence, how that individual incorporates the PERS into his/her daily context will be impacted. This would be an interesting avenue for future PERS research.

#### Theoretical conclusion 3

From the central emergent theme of the decision-making process, it is proposed that in some cases, the intervening conditions in the decision-making process may actually delay or hinder emergency help from being called. This proposition could have larger implications if in very serious emergencies individuals are still hesitant to press, delaying potentially necessary medical or other emergency aid.

### Study limitations

PERS devices usually have a monthly cost associated with them, so seniors who do not have income available for this service were not included. Also the focus of the current study was on community dwelling elderly in an urban setting. Other populations who live at-risk were not included in the present study.

## Conclusions

This grounded theory study found a rich context of independent living for seniors over the age of 80, against a backdrop of unpredictability. PERS allow subscribers to get help if they need it, which provides subscribers with a sense of security or peace of mind. Deciding to subscribe to a PERS and living with the device were both influenced by the perceived risk or unpredictability of daily life of the individual in advanced age. Living with the PERS is structured by routines around when to wear it, where to wear it and keeping the system working, while the main theme of independent living was control over daily life. The decision-making process that emerged fits the role of a cognitive appraisal as part of a problem-focused coping response. The decision-making process involves several considerations, which participants described going through mentally before deciding to activate or not activate their device. Seriousness of the situation, availability of other emergency options, acceptability of the current situation for activating, and self-diagnosis were all part of the process. The way in which participants went through the process and made the decision whether to activate has consequences for daily life with a PERS, and how the participant would choose to use their PERS in the future.

Another avenue for future PERS research would be sampling among high-risk populations, such as individuals in high crime areas or women with high-risk pregnancies to lend more depth to the theme of unpredictability. Also, establishing which influences of daily life are correlated with which decision-making components would shed light on the theoretical model, and create a profile of who is most likely to activate their PERS. Looking at the consequences of pushing and not pushing, such as health outcomes, recovery, PERS response time and the influences of those outcomes on daily life would also help to build the theoretical model surrounding PERS use.

## Abbreviations

PERS: Personal emergency response systems
NORC: Naturally occurring retirement community
